# Herbicides Harm Key Orchard Predatory Mites

**DOI:** 10.3390/insects14050480

**Published:** 2023-05-19

**Authors:** Paul Bergeron, Rebecca Schmidt-Jeffris

**Affiliations:** 1 Department of Entomology, Washington State University, Pullman, WA 99164, USA; 2 Temperate Tree Fruit and Vegetable Research Unit, USDA-ARS, Wapato, WA 98951, USA

**Keywords:** *Galendromus occidentalis*, *Amblydromella caudiglans*, phytoseiid, herbicides, adjuvant, pesticide selectivity, integrated pest management, biological control

## Abstract

**Simple Summary:**

Conserving predators of orchard pests is a critical part of pest management. To achieve this, growers can choose pesticides that are minimally harmful to these predators. While this information is commonly available for insecticides, there is little information about how herbicides affect pest predators. Knowing which herbicides are harmful to predators is particularly important as growers move away from using glyphosate due to consumer safety concerns. Adjuvants are chemicals that are sometimes added to pesticides to increase their efficacy, and their effects on predators are also poorly described. In apple orchards in Washington State, U.S.A., two species of predatory mites are known to be critical for maintaining pest mite populations below damaging levels. We tested seven pesticides and five adjuvants for effects on these important mite predators in laboratory assays. We found that three herbicides (glufosinate, paraquat, and oxyfluorfen) either killed the adult predators or reduced their reproduction. The adjuvants were minimally harmful to the predator mites. Because glufosinate and paraquat are likely replacements for glyphosate, pest mite outbreaks in orchards could result from their increased use.

**Abstract:**

The phytoseiid mites *Galendromus occidentalis* and *Amblydromella caudiglans* are critical for conservation biological control of pest mites in Washington State, U.S.A. apples. While the non-target effects of insecticides on phytoseiids are well described, research on herbicide effects is limited. Using laboratory bioassays, we examined lethal (female mortality) and sublethal (fecundity, egg hatch, larval survival) effects of seven herbicides and five adjuvants on *A. caudiglans* and *G. occidentalis*. The effects of mixing herbicides with recommended adjuvants were also tested to determine if the addition of an adjuvant increased herbicide toxicity. Glufosinate was the least selective herbicide tested, causing 100% mortality in both species. Paraquat caused 100% mortality in *A. caudiglans* and 56% mortality in *G. occidentalis*. Sublethal effects were significant for both species when exposed to oxyfluorfen. Adjuvants did not cause non-target effects in *A. caudiglans*. The non-ionic surfactant and methylated seed oil increased mortality and decreased reproduction in *G. occidentalis*. The high toxicity of glufosinate and paraquat for both predators is concerning; these are the primary “burn down” herbicide alternatives to glyphosate, which is decreasing in use due to consumer toxicity concerns. Field studies are needed to determine the extent to which herbicides disrupt orchard biological control, focusing on glufosinate, paraquat, and oxyfluorfen. Consumer preferences will need to be balanced with natural enemy conservation.

## 1. Introduction

The predatory mites *Galendromus occidentalis* (Nesbitt) and *Amblydromella caudiglans* (Schuster) are the most important and abundant predators of spider mites in Washington State apples [1]. These predators have different life history characteristics, habitat preferences, and prey preferences, providing Washington State apple growers with slightly different biocontrol services [1,2,3,4,5,6]. Specifically, *A. caudiglans* is a generalist predator capable of feeding on tetranychids, eriophyids, and pollen, with a preference for non-web-spinning tetranychids [4,7]. *Galendromus occidentalis* is a more specialized predator of web-spinning tetranychid spider mites (especially *Tetranychus* spp.), although it will also consume other tetranychid species and eriophyids [4]. Prior research indicates that *G. occidentalis* and *A. caudiglans* have similar pesticide sensitivity, although *A. caudiglans* is more sensitive to older, broad-spectrum insecticides [5,8,9]. Conservation of these native predatory mites is based around the judicious use of pesticides for the control of major pests, such as codling moth (*Cydia pomenella* L.). Pesticides are chosen that are minimally harmful to *G. occidentalis*. This has been a cornerstone of integrated mite management since the 1960s [10]. The role of *A. caudiglans* has become appreciated more recently and work is still needed to determine how to best conserve this phytoseiid [1,5].

Across cropping systems, the non-target impacts of herbicides on beneficial arthropods have been ignored [11,12,13]. Although phytoseiids are well represented in pesticide selectivity studies [14], recent reviews have found only a limited number of studies examining the effects of herbicides on this group [12,15]. These reviews indicated that herbicides have the potential to be just as toxic as insecticides, depending on the active ingredient tested [12]. Predatory mites in orchards are known to benefit from ground cover because it provides shelter, floral resources, and alternative prey [16,17,18,19]. However, the negative impacts of bare ground may also be due to the harmful effects of herbicide residues. To date, only one study has examined how in-field herbicide applications impact orchard natural enemies. Applications of paraquat reduced abundance of the phytoseiid *Neoseiulus fallacis* (Garman) and resulted in subsequent spider mite outbreaks [20].

There have been no studies on how herbicides impact either *G. occidentalis* or *A. caudiglans* [12,15]. However, there are opportunities for these phytoseiids to encounter herbicides in the field. *Amblydromella caudiglans* has been found to move between the canopy and the ground cover throughout the season and individuals collected from the canopy have been found with DNA from ground cover plants in their guts, likely from pollen consumption (Bergeron, unpublished). Additionally, higher populations of *A. caudiglans* are associated with orchards with weedy herbicide strips (area directly under the trees) [1]. Similar studies have not been performed for *G. occidentalis*, but individuals have been found adjacent to orchards overwintering on common mullein (*Verbascum thapsus* L.) [21], indicating that it may also move up and down the canopy. It is also plausible that, as prey resources (i.e., spider mites such as *Tetranychus urticae* Koch) move from the canopy to ground cover weeds, these specialized predators would follow.

Most herbicide labels recommend mixing with one or more adjuvants. In Washington, adjuvants are considered pesticides (Washington State Legislature: WAC 16-228-1400). How adjuvants affect pesticide toxicity for natural enemies is virtually unknown. However, work on *Pardosa* spiders has demonstrated that adjuvants can decrease predatory activity when applied alone [22] or in a mixture with glyphosate [23]. Given the potential of adjuvants to increase the harm of herbicides and other pesticides to natural enemies, this line of research should be further investigated to improve conservation recommendations. This is particularly true for the phytoseiids; many adjuvants are oils, which are known to have at least minor non-target effects on predatory mites [24,25].

Given the uncertain future of glyphosate [26,27], research on non-target effects of herbicides is timely. Tree fruit growers in the northwestern U.S.A. are indicating that glufosinate will be their main glyphosate replacement product, but paraquat is also still available and has similar post-weed emergence burn-down. This is concerning for orchard conservation biological control because all current studies on glufosinate suggest it is much more harmful to natural enemies than glyphosate [13,15,28,29]. Paraquat can also cause substantial mortality in a variety of natural enemies [30,31,32,33]. Therefore, if glyphosate is phased out, there is potential for a shift to the use of herbicides, such as glufosinate and paraquat, that are disruptive to biological control of mites and other pests.

For Washington State apple growers, conservation of natural enemies may require judicious herbicide use. The purpose of this study was to determine the lethal and sublethal effects of freshly dried residues of common orchard herbicides and adjuvants independently, as well as the manufacturer’s recommended tank mixtures, on *G. occidentalis* and *A. caudiglans*. The results will be used to determine herbicide use recommendations for conserving these important mite predators.

## 2. Materials and Methods

### 2.1. Study Organisms

*Amblydromella caudiglans* were obtained from a research apple orchard in Moxee, WA (GPS 46.49°, −120.17°) in July–August 2020. Groups of individuals (5–10 adults) were placed in rearing arenas consisting of a single mature lima bean leaf (*Phaseolus lunatus* L. ‘Henderson Bush’; Johnny’s Selected Seeds, Winslow, ME, USA) positioned with its abaxial side up in moistened cotton in a 437 mL plastic cup (APCTR16; Waddington North America, Chattanooga, TN, USA). Wet cotton was pulled up around the edges of the leaf to prevent mites from dispersing from the leaf, and cotton was moistened with water as needed. Mites were provided with a mixed pollen diet of *Typha* spp. (Nutrimite; Biobest, Romulus, MI, USA), apple (*Malus domestica* Borkh.) (‘Regular Strength’ Firman Pollen, Yakima, WA, USA), and *Delosperma cooperi* (Hook.f.) L. Bolus (Family Aizoacae) (grown on site at USDA-ARS, Temperate Tree Fruit and Vegetable Crop Research Unit, Wapato, WA, and pollen collected by hand). Pollen was provided ad libitum weekly using a small paintbrush. A small tuft of cotton was placed on each leaf to serve as an ovipositional substrate. When the density of mites reached 40–50 mites per leaf, the cotton tuft containing eggs along with 5–10 gravid females were transferred to new rearing arenas. The mite cultures were maintained in a growth chamber set at 22 °C, 16:8 [L:D], and all subsequent experiments were held at these settings. A subsample of 10 adult female mites was slide-mounted and identified [34] to confirm species identity after the colony was established.

*Galendromus occidentalis* were purchased from Biotactics (Romoland, CA, USA) and used within 6 h of arrival. A subsample of 10 adult female mites was slide-mounted to confirm the species [34]. *Tetranychus urticae* provided as a food source to *G. occidentalis* during experiments were sourced from a colony maintained at the USDA-ARS (Wapato, WA, USA), which originated from a colony maintained by Cornell University (Geneva, NY, USA). The *T. urticae* were maintained on whole lima bean plants, with half of the plants replaced approximately once weekly.

### 2.2. Predatory Mite Bioassays

Bioassay arenas were created by placing a 1.3 cm diameter bean leaf disk abaxial surface up on moistened cotton inside a 93 mL plastic portion cup. The pesticide (herbicide or adjuvant) concentration used was based on the maximum label rate of the product for apple (Table 1 and Table 2). All pesticide solutions were made by mixing the appropriate amount of formulated product with water to make a 100 mL solution that corresponded to the maximum field rate labelled on apples, applied at 187 L/ha (20 gallons/acre). Pesticide solutions were applied to leaf disks using a laboratory sprayer (Potter Spray Tower, Burkard Scientific, London, UK). Each leaf disk was sprayed with 2 mL of pesticide solution (Table 1) or water (the control) at ~38 kPa and allowed to air dry for ~2 h.

After air-drying, food was added to each arena. *Amblydromella caudiglans* was fed the mixed pollen diet described above by adding a small amount of all three pollens to each disk with a paintbrush. Care was taken to ensure that only a fine, well-spaced dusting of pollen occurred so that the pollen would not serve as a pesticide refuge. Arenas were then allowed to sit overnight. For *G. occidentalis* assays, 10 female *T. urticae* were added to the arena and allowed to oviposit overnight. We confirmed that each arena had >30 *T. urticae* eggs prior to adding a *G. occidentalis* female the next day. The female spider mites were left on the leaf disks to continue laying eggs. Using a fine brush, additional eggs were added as needed from the *T. urticae* colony to provide adequate food for the duration of the experiment (always in excess of 30 eggs). This ensured all individuals had an adequate prey supply throughout the course of the experiment.

Approximately 24 h after pesticide application, a single, visibly gravid *A. caudiglans* or *G. occidentalis* female was added to each arena. Gravid females are the largest individuals within the colony and expansion of the abdomen when an egg is present is noticeable. Females of unknown age were used, as this has not been found to alter the results of pesticide non-target effect studies when gravid individuals are selected [35]. For each treatment tested, there were 30 replicates (individual females), with the exception of testing adjuvants alone on *G. occidentalis*, which used 28 replicates due to limited availability of adult females at that time. Arenas were held in a growth room at ~24 °C and 16:8 photoperiod. After spending 48 h on treated leaf disks, phytoseiid females were recorded as alive or dead and the number of eggs laid were counted. Then, the female was removed from the arena and egg hatch was monitored daily. If a female could not be found, data for that replicate were excluded from the analysis, which is reflected in *n* < 30 or 28 in the results tables. Assays continued until egg hatch in the control reached ~100% hatch, which occurred within 5–8 days. Upon egg hatch, the number of hatched and unhatched eggs, and live larvae were counted in all treatments. Three separate bioassays were conducted for each phytoseiid species: (1) comparison of individual herbicides, (2) comparison of individual adjuvants, and (3) comparison of herbicide and adjuvant mixes. Each bioassay also included a water-sprayed control. For the third assay, only mixes specifically recommended by the herbicide label were examined.

### 2.3. Statistical Analysis

For each bioassay, the data were analyzed separately for each species using a generalized linear model (PROC GLIMMIX, SAS 9.4). Within the bioassay examining herbicide and adjuvant mixes, each mix was compared to a specific herbicide alone to determine if the adjuvant(s) altered the toxicity of the herbicide.

For all analyses, mortality and percentage egg hatch were analyzed using a binomial distribution (dead females/total females or hatched/total eggs) and the number of eggs per live female (fecundity) and total live larvae produced were analyzed using a negative binomial distribution (continuous count data). Replicates where the female was dead at 48 h were not included in the fecundity analysis, but these eggs were used to evaluate percentage hatch. This was performed to avoid confounding female death (no oviposition after the individual died) with actual reproductive effects. If any significant effect was seen (*p* < 0.05), pairwise likelihood ratio contrasts with least-squared means (Tukey adjustment) were used to determine differences between treatments.

To make comparisons between species, data collected in the assays testing herbicides and adjuvants alone were corrected relative to their own control [36]. This allowed for visual comparison between treatments and predator species. Percentage increase (mortality) and percentage decrease (fecundity, egg hatch, live larvae) relative to the control were categorized as low (*x* < 25%), moderate (25% ≥ *x* < 75%), and high (*x* ≥ 75%) toxicity [37].

## 3. Results

### 3.1. Herbicides Alone

Glufosinate and paraquat residues resulted in 100% mortality in *A. caudiglans* females and were the only herbicides to differ from the water control (Table 3). Consequently, these treatments resulted in no egg production and were excluded from the fecundity analysis. Only oxyfluorfen was found to significantly reduce egg production (61% reduction from the control). Oxyfluorfen also reduced egg hatch (9%), as did 2,4-D (78%) (Table 3). Glufosinate, paraquat, and oxyfluorfen had the lowest production of *A. caudiglans* live larvae (0) and 2,4-D had an intermediate effect compared to these herbicides and the control (56% reduction).

Glufosinate also caused 100% mortality in *G. occidentalis*, but paraquat was intermediately harmful (57% mortality) (Table 4). Paraquat and oxyfluorfen caused the greatest reduction in fecundity, followed by 2,4-D. All other herbicides were similar to the control. Of the herbicides where sufficient eggs were laid for analysis (which excluded glufosinate and paraquat), only oxyfluorfen was found to reduce egg hatch (Table 4). Live larvae production was the lowest in the glufosinate, paraquat, and oxyfluorfen treatments. Halosulfuron, glyphosate, and 2,4-D also had fewer live larvae than the control (Table 4).

### 3.2. Adjuvants Alone

None of the adjuvants had significant non-target effects on *A. caudiglans* (Table 5). However, the non-ionic surfactant caused numeric reductions in fecundity, egg hatch, and larvae production. AMS caused a numeric decrease in fecundity and live larvae production (Table 5).

For *G. occidentalis*, NIS and MSO caused a reduction in fecundity compared to the control, which also resulted in significantly decreased production of live larvae (Table 6). Additionally, NIS, MSO, and COC also caused a numeric increase in mortality (21–29% versus 4% in the control).

### 3.3. Herbicide and Adjuvant Mixes

In *A. caudiglans*, addition of adjuvants did not change any of the parameters measured relative to the herbicide alone (Table 7). This was also generally true for *G. occidentalis*, except that the addition of NIS to 2,4-D caused a reduction in fecundity from 2.00 eggs per female to 1.36 eggs per female (Table 8). However, this is not much different from the control (1.53 eggs/female). Therefore, this study did not find substantial evidence that adjuvants increase non-target effects of herbicides on predatory mites.

### 3.4. Relative Toxicity Ratings

**Figure 1:**
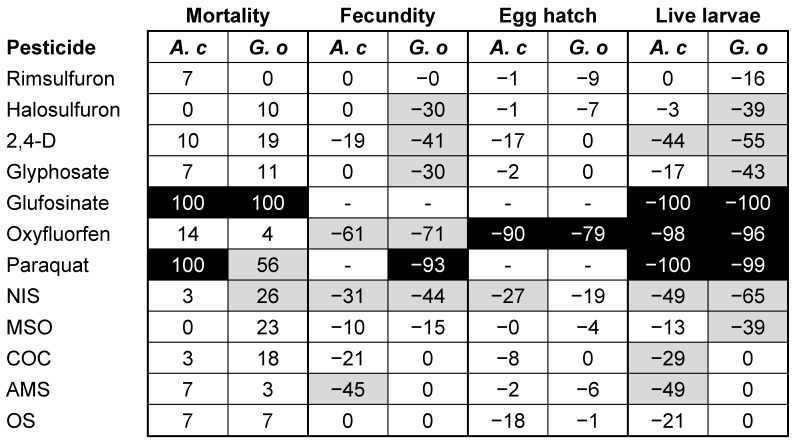
Summary of lethal and sublethal effects based on corrected values, showing percent increase (mortality) or percent decrease (all other) in comparison to the control. Values are categorized as low (*x* < 25%, white), moderate (25% ≥ *x* < 75%, light gray), and high (*x* ≥ 75%, black) [37]. When a treatment resulted in less mortality or greater fecundity, egg hatch, or live larvae than the control, the corrected value was set to “0”.

Relative to the control, both mites were fairly similar in mortality across all materials tested (Figure 1). Paraquat was moderately harmful to *G. occidentalis* but highly harmful to *A. caudiglans*. In general, herbicides caused greater reductions in fecundity for *G. occidentalis* than *A. caudiglans*. This resulted in halosulfuron and glyphosate being rated as “intermediate” for *G. occidentalis* but “low” for *A. caudiglans* (Figure 1). In the case of oxyfluorfen, the “highly harmful” rating for both species was due to a combination of reduced fecundity and egg hatch. Finally, due to reduced fecundity, halosulfuron and glyphosate were rated as intermediate for *G. occidentalis* but low for *A. caudiglans*.

None of the adjuvants was rated as highly harmful to either mite (Figure 1). Based on larvae production, *Galendromus occidentalis* appeared to be slightly more sensitive to MSO, whereas *A. caudiglans* was more sensitive to COC and AMS. Between both predators, NIS was the most harmful adjuvant (Figure 1).

## 4. Discussion

Herbicides clearly have the potential to disrupt biological control provided by predatory mites in apple orchards. Mortality caused by glufosinate and paraquat is comparable to that of broad-spectrum insecticides that are known to cause spider mite outbreaks in orchards [5,37]. Additionally, the sublethal effects of oxyfluorfen resulted in almost no viable offspring for treated females. However, there is the potential that “wild” strains of *G. occidentalis* would respond to herbicides differently than the insectary population tested in this study, or that other populations of *A. caudiglans* would respond differently. The insectary strain in particular may be more susceptible to pesticides in general than a field population. Therefore, this study only reflects first steps towards determining how herbicide applications will impact predatory mites in the field. Impacts of glufosinate, paraquat, and oxyfluorfen in particular should be further examined for their potential to increase pest mite damage in the field. These results are particularly concerning given that glufosinate and paraquat are the primary post-emergent alternatives to glyphosate. Because glyphosate resulted in little mortality and less substantial sublethal effects for both predators, increased use of other herbicides as a result of reduced glyphosate use is a probable concern for biological control.

The non-target effects of glufosinate on natural enemies in general are poorly understood, relative to other pesticides [38]. The phytoseiids *Phytoseiulus persimilis* Athias-Henriot, *N. fallacis*, and *Amblyseius womersleyi* Schicha experienced significant increases in mortality following treatment with glufosinate [28,29,39]. In all studies on phytoseiids and glufosinate, the herbicide has been found harmful to the phytoseiid tested [15]. In other natural enemies, results have been more varied. Little mortality was seen in *Chrysopa pallens* Rambur or adult *Harmonia axyridis* (Pallas) [28]. However, glufosinate caused substantial mortality in *Orius strigicollis* Poppius and some juvenile stages of *H. axyridis* [28]. In the parasitoid *Palmistichus elaeisis* Delvare and LaSalle, glufosinate treatment reduced parasitism and emergence rates [40]. In three species of spiders found in orchards, exposure to glufosinate residues did not cause any mortality [13], but direct application increased mortality in *Pardosa agrestis* (Westring) [38]. The impact of glufosinate on a greater variety of natural enemies needs to be examined in the lab and the field, but the current trend indicates that glufosinate use could be particularly disruptive to integrated mite management.

Of the tested herbicides, paraquat is the only one to have been directly linked to outbreaks of secondary pests under field conditions [20]. This has been attributed to its non-target effects on *N. fallacis* [30,39,41]. Laboratory studies on *Euseius hibicsci* (Chant) also found that paraquat caused ~50% mortality and low fecundity when females were exposed to dry residues [32]. While *N. fallacis* is the primary predator of spider mites in northeastern U.S. orchards, this role is filled by *G. occidentalis* and *A. caudiglans* in the northwest [42]. Therefore, there is a significant risk that paraquat use is equally harmful to northwestern mite biological control. Given this and the substantial human health risks of paraquat [43], its use should be highly limited.

Although oxyfluorfen caused little direct mortality, significant sublethal effects on *A. caudiglans* and *G. occidentalis* were observed. Accumulation of sublethal effects via reduced fecundity and egg hatch reduced production of live larvae by treated females to nearly zero. Previous research on non-target effects of oxyfluorfen to *N. fallacis* have reported high mortality (96%) [39], while work on *P. persimilis* suggests moderate toxicity (33–36% mortality) [15]. Neither of these studies examined reproductive effects. Interestingly, various orchard-inhabiting spiders experienced relatively highly mortality after 48 h of exposure to residues, even though they had very little susceptibility to other herbicides tested, including paraquat and glufosinate [13]. How oxyfluorfen affects natural enemy populations in orchards merits further study.

In general, 2,4-D has been found to be harmless to phytoseiids [15], including *P. persimilis*, *Amblyseius andersoni* (Chant), and *Typhlodromus pyri* Scheuten [29,44]. However, a study examining the effects of 2,4-D on *N. fallacis* and *T. urticae* found that it was over 4× as toxic to the predator than the pest, suggesting the potential for biological control disruption [45]. Our study found that 2,4-D primarily impacts phytoseiids through reduced fecundity and egg hatch. Further studies on laboratory non-target effects and follow-up semi-field and field studies are needed to determine if 2,4-D alters predator:prey ratios.

Glyphosate is among the most studied herbicides due to growing consumer concerns about its overuse in agricultural and urban landscapes. For phytoseiids, impacts of glyphosate in laboratory studies have been highly variable [15] based on both the species and formulation tested, with findings of both “highly toxic” [32,41] and “harmless” [46]. Species that are typically considered more susceptible to pesticides (*Euseius* spp., *N. fallacis*) were more sensitive to glyphosate, and those that are known to be less susceptible (*P. persimilis*, *T. pyri*) were those in the “harmless” category [12,15]. Given the extreme number of glyphosate formulations available [47], it is not surprising that toxicity to phytoseiids would be equally variable. In honey bees, glyphosate as pure AI has been found to be non-toxic, but many of the surfactants and other additives in the formulation have known non-target effects [47]. Because the present study and previous work have shown that glyphosate is less toxic to natural enemies than some of its alternatives, identifying formulations of glyphosate that are minimally harmful to beneficial insects and human health [47] may be essential for successful IPM.

Few studies have examined non-target impacts of rimsulfuron and halosulfuron, which are both sulfonylurea herbicides. In one study, halosulfuron did not increase *P. persimilis* mortality compared to the control when exposed by direct contact or residues [15]. In spiders, neither herbicide caused any mortality even after five days of exposure to residues [13]. However, both herbicides appeared to be an irritant to *Philodromus cespitum* (Walckenaer), increasing its movement by over five-fold [13]. Both herbicides also reduced prey consumption by *Pelegrina aeneola* (Curtis) [13]. There are no other studies in the literature on either halosulfuron’s or rimsulfuron’s non-target effects. However, research on other sulfonylureas indicates this group is among the least harmful herbicides to natural enemies [48,49,50,51].

Non-target effects of adjuvants on *G. occidentalis* and *A. caudiglans* were minimal. This study suggests that the non-ionic surfactant and methylated seed oil have the greatest impact on these two species, although significant effects were only observed in *G. occidentalis*. These adjuvants are commonly referred to as activator compounds because they are designed for spreading, dispersion, emulsification, and increased penetration of the plant leaf surface to improve herbicide activity [52,53,54,55]. A possible explanation for the sublethal effects due to exposure with adjuvant may be found in the viscosity of methylated seed oil or the non-ionic surfactant; oils are known to be harmful to predatory mites. While adjuvants are not technically pesticides, some states within the U.S.A. (e.g., Washington) consider them pesticides for regulatory purposes. Unfortunately, there is very little research on the non-target effects of adjuvants on natural enemies, limiting our ability to confirm results from the present study. Given the research indicating that supposed inert ingredients can significantly harm beneficial insects [23,47], pesticide testing should increase its focus to also examine differences in formulation toxicity.

The mechanisms of toxicity for non-insecticides on non-target arthropods are virtually unstudied and the mode of action of herbicides on arthropods remains unknown [56]. There are no consistent patterns regarding toxicity within a herbicide mode of action group [14,15]. Toxicity may be linked to the physical properties of the active ingredients, such as lipophilicity or volatility [15]. In one study examining *T. urticae* and *P. persimilis*, inactive ingredients within the formulated product caused high viscosity, resulting in smothering of both species after contact exposure [15]. This effect was not seen when the mites were exposed to residues [15]. Glyphosate has been found to alter the endosymbiont community within a ladybeetle, and these changes were associated with reduced body weight [57]. In a ground beetle, exposure to the herbicide pendimethalin resulted in decreased gut microbiome diversity and decreased abundance of various bacteria genera associated with metabolism and detoxification [58]. Many additional toxicology studies will be necessary to determine how herbicide active ingredients and formulations cause non-target effects in arthropods.

In an orchard, the two predatory mites might differ in their potential for exposure to herbicides. In surveys of pear orchards, *A. caudiglans* is more commonly found in the ground cover than *G. occidentalis* (Schmidt-Jeffris, unpublished), increasing its exposure risk. Host plant preferences may also affect herbicide exposure. *Amblydromella caudiglans* appears to prefer host plants with trichome-dense leaves [1,59], potentially because the trichomes serve as pollen traps and *A. caudiglans* is a generalist phytoseiid that can sustain population growth on pollen diets [7,60,61]. Trichomes can reduce pesticide penetration [55], which could offer *A. caudiglans* some protection on these leaf surfaces. There is evidence that *Galendromus occidentalis* may also prefer trichome-dense host plants [59], but other studies have suggested that this more specialist species may not have strong host plant preferences [1,62]. Multiple aspects of phytoseiid biology might alter how these predators respond to herbicides in the field.

Because our study used laboratory assays, it can only identify potentially harmful herbicides. Field studies are needed to confirm the effects of these herbicides on phytoseiid populations in orchards and to determine if the effects are significant enough to cause pest outbreaks. There are virtually no field studies examining how herbicide applications for weed management in crops impact natural enemy abundance and biological control services. This is likely because impacts of herbicide toxicity and loss of habitat, floral resources, and alternative prey provided by weeds are difficult to detangle. Because reducing weed management has been associated with increased phytoseiid abundance and biological control [16,17,18,19], the effects of various weed management practices should be tested to find combinations that reduce impact on natural enemies while still optimizing yield. Judicious weed control may be particularly important for conserving *A. caudiglans* [1]. Therefore, the creation of a resilient natural enemy community must incorporate alterations to how orchard ground cover is managed.

## Figures and Tables

**Table 1:** Herbicides used in non-target effects bioassays.

Active Ingredient	Mode of Action ^1^	Brand Name	Use Rate (g AI/ha)	Mix (Amount Product/L Solution)
Rimsulfuron	2	Dupont Matrix SG ^2^	70	1.50 g
Halosulfuron	2	Sandea ^3^	105	0.75 g
2,4-D	4	Weedar 64 ^4^	218	25.00 mL
Glyphosate	9	Glyphosate-4DS ^5^	1112	12.50 mL
Glufosinate-ammonium	10	Cheetah ^4^	1680	32.03 mL
Oxyfluorfen	14	Goal 2XL ^4^	2242	50.00 mL
Paraquat	22	Gramoxone SL 2.0 ^6^	1548	25.00 mL

^1^ Mode of action group, as defined by the Herbicide Resistance Action Committee. ^2^ Corteva AgriScience, Wilmington, DE, USA. ^3^ Gowan Company LLC, Yuma, AZ, USA. ^4^ Nufarm Americas Inc., Alsip, IL, USA. ^5^ Genesis Agri Products Inc., Union Gap, WA, USA. ^6^ Syngenta Crop Protection, Inc., Greensboro, NC, USA.

**Table 2:** Adjuvants used in non-target effects bioassays.

Type of Adjuvant	Brand Name	mL Adjuvant/L Solution
Non-ionic Surfactant (NIS)	Nu-Film ^1^	1.25
Crop Oil Concentrate (COC)	Crop Oil Concentrate ^2^	10
Ammonium Sulfate (AMS)	Titan Spray Grade AMS ^3^	3.59
Methylated Seed Oil (MSO)	Methylated Seed Oil Surfactant ^4^	10
Organosilicone (OS)	Kinetic ^2^	5

^1^ Miller Chemical & Fertilizer, LLC, Hanover, PA, USA. ^2^ Helena Agri Enterprises LLC, Collierville, TN, USA. ^3^ Titan Ag Pty, Belrose, NSW, Australia. ^4^ Loveland Products, Inc., Greeley, CO, USA.

**Table 3:** Mortality (uncorrected), fecundity (eggs laid/live female), egg hatch, and larval production of *A. caudiglans* treated with herbicides as adult females (mean ± SEM). Values within columns followed by the same letter are not significantly different (LSMEANS, *p* < 0.05).

Herbicide	*n*	% Mortality	Eggs/Live Female	*n* Eggs	% Egg Hatch	Live Larvae/Female
Rimsulfuron	30	10 b	1.52 ± 0.22 a	41	92.5 ± 5.5 ab	1.27 ± 0.22 a
Halosulfuron	30	0 b	1.30 ± 0.23 a	39	92.5 ± 5.5 ab	1.17 ± 0.20 ab
2,4-D	30	13 b	1.00 ± 0.23 a	28	77.8 ± 10.6 b	0.67 ± 0.19 b
Glyphosate	30	10 b	1.30 ± 0.21 a	35	91.2 ± 4.1 ab	1.00 ± 0.18 ab
Glufosinate	29	100 a	-	0	-	0 ± 0 c
Oxyfluorfen	30	17 b	0.48 ± 0.12 b	12	9.1 ± 9.1 c	0.03 ± 0.03 c
Paraquat	20	100 a	-	0	-	0 ± 0 c
Control	30	3 a	1.24 ± 0.20 a	38	93.2 ± 5.0 a	1.20 ± 0.19 a
*F*		6.81	2.74		4.80	5.96
df		7, 221	5, 158		5, 101	7, 232
*p*		<0.0001	0.0210		0.0006	<0.0001

**Table 4:** Mortality (uncorrected), fecundity (eggs laid/live female), egg hatch, and larval production of *G. occidentalis* treated with herbicides as adult females (mean ± SEM). Values within columns followed by the same letter are not significantly different (LSMEANS, *p* < 0.05).

Herbicide	*n*	% Mortality	Eggs/Live Female	*n* Eggs	% Egg Hatch	Live Larvae/Female
Rimsulfuron	30	0 c	2.40 ± 0.18 a	72	87.1 ± 4.0 a	1.87 ± 0.18 ab
Halosulfuron	30	13 c	1.69 ± 0.23 ab	50	88.6 ± 5.3 a	1.37 ± 0.21 bc
2,4-D	29	21 c	1.43 ± 0.27 b	33	96.4 ± 2.5 a	1.00 ± 0.2 c
Glyphosate	29	14 c	1.68 ± 0.24 ab	43	98.3 ± 1.7 a	1.27 ± 0.22 bc
Glufosinate	30	100 a	-	2	0 ± 0 *	0 ± 0 d
Oxyfluorfen	30	7 c	0.71 ± 0.14 c	20	20.0 ± 10.7 b	0.10 ± 0.07 d
Paraquat	28	57 b	0.17 ± 0.11 c	2	50 ± 50 *	0.03 ± 0.03 d
Control	30	3 c	2.41 ± 0.18 a	70	95.7 ± 2.5 a	2.23 ± 0.20 a
*F*		3.80	7.05		8.34	11.49
df		7, 228	6, 166		5, 122	7, 232
*p*		0.0006	<0.0001		<0.0001	<0.0001

* Excluded from analysis due to <3 eggs (replicates).

**Table 5:** Mortality (uncorrected), fecundity (eggs laid/live female), egg hatch, and larval production of *A. caudiglans* treated with adjuvants as adult females (mean ± SEM).

Adjuvant	*n*	% Mortality	Eggs/Live Female	*n* Eggs	% Egg Hatch	Live Larvae/Female
NIS	30	3	0.90 ± 0.16	26	70.6 ± 10.6	0.63 ± 0.15
MSO	30	0	1.17 ± 0.12	34	96.0 ± 4.0	1.07 ± 0.13
COC	30	3	1.03 ± 0.13	30	89.1 ± 6.3	0.87 ± 0.12
AMS	30	7	0.71 ± 0.11	20	94.4 ± 5.6	0.63 ± 0.11
OS	30	7	1.36 ± 0.16	38	79.2 ± 7.9	0.97 ± 0.17
Control	30	0	1.30 ± 0.11	39	96.4 ± 3.6	1.23 ± 0.11
*F*		0.14	1.59		2.05	1.87
df		5, 174	5, 168		5, 129	5, 174
*p*		0.9840	0.1660		0.0763	0.1027

**Table 6:** Mortality (uncorrected), fecundity (eggs laid/live female), egg hatch, and larval production of *G. occidentalis* treated with adjuvants as adult females (mean ± SEM). Values within columns followed by the same letter are not significantly different (LSMEANS, *p* < 0.05).

Adjuvant	*n*	% Mortality	Eggs/Live Female	*n* Eggs	% Egg Hatch	Live Larvae/Female
NIS	28	29	1.40 ± 0.33 c	28	80.8 ± 10.6	0.82 ± 0.24 b
MSO	27	26	2.10 ± 0.34 bc	42	96.4 ± 2.5	1.43 ± 0.29 b
COC	28	21	3.20 ± 0.35 a	71	100 ± 0	2.36 ± 0.38 a
AMS	27	7	3.16 ± 0.27 a	79	93.9 ± 4.6	2.64 ± 0.31 a
OS	28	11	3.24 ± 0.23 a	81	98.9 ± 1.1	2.86 ± 0.28 a
Control	28	4	2.48 ± 0.36 ab	67	100 ± 0	2.36 ± 0.35 a
*F*		1.84	4.72		1.61	5.66
df		5, 160	5, 133		5, 107	5, 162
*p*		0.1082	0.0005		0.1645	<0.0001

**Table 7:** Mortality (uncorrected), fecundity (eggs laid/live female), egg hatch, and larval production of *A. caudiglans* treated with mixtures of herbicides and label-recommended adjuvants as adult females (mean ± SEM). * Treatment results identical, no statistical analysis.

Treatment	*n*	% Mortality	Eggs/Live Female	*n* Eggs	% Egg Hatch	Live Larvae/Female
Rimsulfuron	29	0	0.41 ± 0.14	12	88.9 ± 11.1	0.37 ± 0.13
Rimsulfuron + NIS	29	10	0.96 ± 0.19	25	93.3 ± 4.5	0.73 ± 0.15
Rimsulfuron + MSO	28	4	0.85 ±0.15	24	100 ± 0	0.73 ± 0.14
Rimsulfuron + COC	29	7	0.67 ± 0.12	18	93.8 ± 6.3	0.53 ± 0.11
*F*		0.31	1.65		0.04	1.35
df		3, 111	3, 105		3, 54	3, 116
*p*		0.8150	0.1815		0.9894	0.2629
Halosulfuron	29	7	1.07 ± 0.18	31	100 ± 0	0.97 ± 0.17
Halosulfuron + NIS	30	0	1.10 ± 0.15	33	100 ± 0	1.00 ± 0.15
*F*		0.00	0.03		*	0.05
df		1, 57	1, 55			1, 58
*p*		0.9765	0.8553			0.8217
2,4-D	30	20	0.75 ± 0.16	18	88.5 ± 8.3	0.53 ± 0.13
2,4-D + NIS	30	0	0.47 ± 0.14	14	90.0 ± 10.0	0.40 ± 0.12
*F*		3.16	1.53		0.14	0.55
df		1, 58	1, 51		1, 21	1, 58
*p*		0.0808	0.2220		0.7084	0.4619
Glyphosate	30	3	0.69 ± 0.13	20	100 ± 0	0.63 ± 0.13
Glyphosate + NIS	30	10	0.63 ± 0.14	17	100 ± 0	0.50 ± 0.12
Glyphosate + AMS	29	10	0.62 ± 0.17	18	88.5 ± 8.3	0.53 ± 0.14
*F*		0.58	0.08		0	0.24
df		2, 86	2, 79		2, 40	2, 87
*p*		0.5619	0.9240		0.9995	0.7842
Glufosinate	28	100	-	0	-	0 ± 0
Glufosinate + AMS	30	100	-	0	-	0 ± 0
		*				*
Control	30	3	1.03 ± 0.20	30	100 ± 0	1.00 ± 0.19

**Table 8:** Mortality (uncorrected), fecundity (eggs laid/live female), egg hatch, and larval production of *G. occidentalis* treated with mixtures of herbicides and label-recommended adjuvants as adult females (mean ± SEM). Values within columns followed by the same letter are not significantly different (LSMEANS, *p* < 0.05). * Treatment results identical, no statistical analysis.

	*n*	% Mortality	Eggs/Live Female	*n* Eggs	% Egg Hatch	Live Larvae/Female
Glufosinate	30	100	-	0	-	0 ± 0
Glufosinate + AMS	30	100	-	0	-	0 ± 0
		*				*
Halosulfuron	30	0	1.27 ± 0.21	38	100 ± 0	1.27 ± 0.21
Halosulfuron + NIS	29	3	1.61 ± 0.19	45	100 ± 0	1.50 ± 0.19
*F*		0.00	1.54		*	0.78
df		1, 57	1, 56			1, 58
*p*		0.9778	0.2196			0.3802
2,4-D	30	0	2.00 ± 0.17 a	60	95.4 ± 4.0	1.93 ± 0.18
2,4-D + NIS	29	3	1.36 ± 0.21 b	38	98.8 ± 1.3	1.23 ± 0.20
*F*		0.00	4.58		0.04	0.55
df		1, 57	1, 56		1, 47	1, 58
*p*		0.9778	0.0368		0.8454	0.4619
Glyphosate	30	7	1.35 ± 0.19	38	98.5 ± 1.5	1.13 ± 0.18
Glyphosate + AMS	30	3	0.79 ± 0.14	24	100 ± 0	0.80 ± 0.14
Glyphosate + NIS	29	7	1.48 ± 0.22	43	88.6 ± 6.5	1.27 ± 0.20
*F*		0.21	2.56		0.68	1.23
df		2, 86	2, 81		2, 56	2, 87
*p*		0.8074	0.0835		0.5088	0.2960
Control	30	0	1.53 ± 0.20	46	99 ± 1	1.50 ± 0.18

## Data Availability

The data presented in this study are available on request from the corresponding author.
